# The Costs and Benefits of Mindfulness and Reappraisal in Daily Life

**DOI:** 10.1007/s42761-022-00178-7

**Published:** 2023-02-20

**Authors:** Mario Wenzel, Elisabeth S. Blanke, Zarah Rowland, Annette Brose

**Affiliations:** 1 Psychologisches Institut, Johannes Gutenberg-Universität Mainz, Binger-Str. 14–16, 55122 Mainz, Germany; 2 Humboldt-Universität zu Berlin, Berlin, Germany; 3 Friedrich-Schiller-Universität Jena, Jena, Germany; 4 German Institute for Economic Research (DIW Berlin), Berlin, Germany

**Keywords:** Reappraisal, Acceptance, Mindfulness, Emotion regulation, Affect

## Abstract

Reappraisal and mindfulness represent two fundamentally different but interconnected ways of dealing with one’s emotions: whereas reappraisal is aimed at changing one’s thoughts and emotions, mindfulness is aimed at *not* immediately changing, but appreciating them. Despite this difference, prior research has shown that both are beneficial for one’s affective well-being. However, research on the spontaneous use of reappraisal and mindfulness in daily life found that they might be differentially associated with positive and negative affect, with reappraisal and mindful attention being more strongly associated with increased positive affect and mindful acceptance with decreased negative affect. Moreover, the spontaneous use of reappraisal may be less effective than mindfulness in daily life given that it is more cognitively taxing. To compare these possibly different benefits (i.e., change in positive and negative affect) and costs (i.e., feeling depleted), we re-analyzed two experience sampling studies (*N* = 125 and *N* = 179). Regarding benefits, endorsing reappraisal and mindful attention was significantly associated with increases in positive affect, whereas endorsing mindful acceptance was significantly associated with decreases in negative affect. Regarding costs, we found that endorsing reappraisal led to more depletion and that reappraisal was selected less often than mindfulness in daily life. Our results demonstrate the importance of assessing not only the different benefits but also the costs of emotion regulation in daily life.

**Supplementary Information:**

The online version contains supplementary material available at 10.1007/s42761-022-00178-7.

Emotions are a vital part of human life that can be considered functional by directing individuals’ behavior (Frijda, 2007). However, despite their general adaptiveness, emotions have to be regulated appropriately by identifying the need for emotion regulation (ER) and by endorsing suitable strategies (Sheppes et al., 2015). Prior research has demonstrated that ER “failures” can negatively impact individuals’ well-being (Aldao et al., 2010), ranging from depression to eating disorders (Berking & Wupperman, 2012) to the regulation of glucose and immune responses (Thayer & Sternberg, 2006).

One of the most elaborately studied ER strategies is reappraisal, a cognitive-change strategy that is aimed at influencing the meaning of an emotion-eliciting stimulus or its context (Gross, 2015). A meta-analysis has found that reappraisal can be viewed as generally adaptive, as indicated by a mean effect size of *d* = 0.38 for the instructed use of reappraisal (Webb et al., 2012).

In the last decade, mindfulness has gained increasing interest as another process that contributes to adaptive ER (Chiesa et al., 2013). Mindfulness has been defined as “paying attention in a particular way: on purpose, in the present moment, and nonjudgmentally” (Kabat-Zinn, 1994, p. 8). Thus, reappraisal and mindfulness represent two fundamentally different approaches to ER, as reappraisal is aimed at changing one’s thoughts and emotions, whereas mindfulness is aimed at *not* immediately changing, but appreciating them. Although reappraisal and mindfulness seem to have opposite goals, research has found that both are associated with adaptive outcomes (Shallcross et al., 2015), with a mean effect size of *d* = 0.31 for the instructed use of mindfulness, although there is a large heterogeneity in the effect sizes (Webb et al., 2012). In the present research, we examined whether this heterogeneity can be explained by the differential benefits and costs of endorsing reappraisal and mindfulness in daily life, using two experience sampling datasets.

## Differential Benefits of Reappraisal and Mindfulness

As a measure of benefits, we focused on increases in self-reported positive affect (PA) or decreases in negative affect (NA) after participants spontaneously endorsed reappraisal and mindfulness in daily life. In accordance with prior research (e.g., Brans et al., 2013), reappraisal and mindfulness are deemed more effective; the stronger the increase in PA or the stronger the decrease in NA is when they are endorsed. Research investigating effectiveness has produced mixed findings. For example, the spontaneous use of cognitive reappraisal (reappraising the cause of affective experiences) was not significantly associated with decreases in current NA (Brans et al., 2013; Wenzel et al., 2020) but only with increases in PA (Brans et al., 2013). In another study, significant associations between spontaneously using positive reappraisal (positively reappraising the situation) and subsequent changes in both PA and NA emerged (Pavani et al., 2017). This indicates that subtle differences between different types of reappraisal may differently be associated with momentary feelings.

Similarly, different conceptualizations of mindfulness lead to different associations with affect. Using a unidimensional measure of mindfulness, research has found positive associations with PA and negative associations with NA (Brown & Ryan, 2003). However, mindfulness can also be conceptualized multidimensionally, based on at least two major components (Bishop et al., 2004): (1) the regulation of attention to the present moment (i.e., mindful attention) and (2) the non-judgmental acceptance of these present-moment experiences (i.e., mindful acceptance).^1^ Such a more fine-grained view on mindfulness revealed that while mindful acceptance was more strongly associated with current NA than mindful attention, it was less strongly associated with current PA, both in daily life (Blanke et al., 2018) and after mindfulness-based cognitive therapy (Schroevers & Brandsma, 2010). Moreover, mindful acceptance was associated with dampened stress reactivity (Blanke et al., 2018; Wenzel et al., 2021), whereas mindful attention was related to PA (Blanke et al., 2018). Thus, different aspects of reappraisal and mindfulness may have different benefits regarding current affective experiences.

Most research directly comparing reappraisal and mindfulness used experimental approaches, which has produced mixed findings, too: out of five studies, three found that the instructed use of reappraisal compared to mindful acceptance was more effective in decreasing NA (Hofmann et al., 2009; Szasz et al., 2011; Troy et al., 2018). Two others did not find any difference (Asnaani et al., 2013; Wolgast et al., 2011). Regarding the spontaneous use in daily life, participants reported more success in decreasing current NA when they endorsed mindfulness compared to cognitive reappraisal, with the latter also not being significantly associated with increased PA in an experience sampling study (Heiy & Cheavens, 2014). Given the scarce evidence, we add to these findings by comparing the benefits of the spontaneous use of reappraisal, mindful attention, and mindful acceptance. We thereby can compare potentially differential affective consequences.

In more detail, we examined both cognitive (Study 1) and positive reappraisal (Study 2) as well as mindfulness unidimensionally (as mindful attention; Study 1) and multidimensionally (Study 2). Replicating past evidence and given the focus of positive reappraisal on PA, we expected reappraisal, especially positive reappraisal, as well as mindful attention to be *more* strongly associated with increased PA compared to mindful acceptance (Hypothesis 1). We also hypothesized that the spontaneous use of reappraisal and mindful attention is *less* strongly associated with decreased NA compared to mindful acceptance (Hypothesis 2).

## Differential Costs of Reappraisal and Mindfulness

Using ER strategies not only comes with benefits, but may also come with costs, specifically in the form of cognitive resource expenditure (Sheppes, 2020), as endorsing ER strategies can be effortful. And, indeed, research has demonstrated such costs: first, reappraisal was found to be selected less often because of its high effort to endorse it (Milyavsky et al., 2019). Second, the choice of reappraisal seems to depend on the intensity of NA or PA in situations: Individuals were more likely to choose reappraisal over distraction in situations characterized by lower levels of NA, with the reversing pattern in high-intensity negative situations (e.g., Sheppes et al., 2011). This effect was attributed to the cognitive costs of reappraisal. Specifically, when strong emotions have already developed, reappraisal requires to override the strong and already established appraisals of the given situation, which is mentally taxing and, thus, costly in terms of cognitive resource expenditure (Sheppes, 2020). Individuals may try to avoid these costs by selecting other, less effortful ways of dealing with their emotions (Milyavsky et al., 2019). Together, although reappraisal is generally beneficial, individuals endorse it less often than would be expected, which has been demonstrated both in the lab (Suri et al., 2015) and in daily life (e.g., Brans et al., 2013; Heiy & Cheavens, 2014).

In contrast to reappraisal, mindfulness should tax cognitive resources less strongly than reappraisal given that it is aimed at not changing one’s impulses (Shallcross et al., 2013). However, evidence for this notion is relatively scarce. Prior laboratory research has shown that practicing mindfulness was not only associated with relaxation (Rosch, 2007) but also with replenished cognitive resources (Friese et al., 2012). Only two laboratory research projects compared the cognitive costs associated with reappraisal and mindfulness: participants in a mindfulness or reappraisal condition in a laboratory experiment involving the Stroop task both reported significantly lower levels of sadness compared to the control condition (Keng et al., 2013). However, participants in the reappraisal condition performed slower on incongruent compared to congruent trials compared to the mindfulness condition, indicating a greater level of mental fatigue. Moreover, reappraisal was associated with significantly larger increases in PA and decreases in NA than mindful acceptance when regulating emotions in reaction to a sad video (Troy et al., 2018). However, participants deemed mindful acceptance as less difficult to endorse than reappraisal.

Thus, we built on this research and examined the costs of endorsing reappraisal and mindfulness in daily life. We tested whether individuals felt less depleted when endorsing mindfulness in comparison to reappraisal (Hypothesis 3). Moreover, we hypothesized that when individuals could choose freely, they often favor mindfulness over reappraisal because the latter should be more difficult and taxing (Hypothesis 4).

## Study 1

### Method

Table 1 provides an overview of the dataset in Study 1.

**Table 1:** Overview of the datasets

Dataset	Study 1	Study 2
Parent study	Rowland et al. (2016)	Blanke et al. (2020)
Type	ESM (self-report)	ESM (self-report)
Country of data collection	Germany	Germany
Participants: *N*	125	179
Gender: % female	77.6%	52.5%
Age: *M* in years (*SD*)	22.9 (5.1)	50.9 (5.8)
Number of days	40	12 (3 times 4 days with 4 pause days in between)
Observations per day	6	6
Max. number of observations	240	96 (goal: 60)
Average no. of observations/participant: *M* (*SD*)	182.9 (35.3)	69.3 (7.6)
ESM application	movisensXS (movisens GmbH, Karlsruhe, Germany)	Custom built
ESM hardware (smartphones)	Motorola Moto G	Huawei Ascend G330
Compensation	Course credits	80–90 €
Adherence	76.2%	96.3%
PA items	Excited, happy, relaxed, satisfied	Content, inspired, interested, joyful, relaxed, well
NA items	Angry, anxious, depressed, sad	Angered, distressed, downhearted, jittery, nervous, upset

*ESM* experience sampling method, *ICC* intraclass correlation, *PA* positive affect, *NA* negative affect

#### Participants

The parent study is aimed at recruiting 137 participants to achieve an a priori power of 95% to detect a small effect of Cohen’s *d* = 0.33 against *α* = 0.05 and an expected attrition of 10% for the difference between the mindfulness and a wait-list control condition in state mindfulness. Thus, data of 137 participants were collected of which 11 participants dropped out during the study and 1 was excluded due to low compliance of fewer than 33% completed signals. No commonly accepted guidelines exist regarding the minimum number of completed observations in experience sampling studies. To be consistent with our prior handling of the datasets, we excluded participants with fewer than 33% completed observations in Study 1 only. This yielded a final sample of *N* = 125 participants (77.6% female; *M* = 22.9 years, *SD* = 5.1), with 64 participants in the control condition and 61 participants in the mindfulness condition.

#### Procedure

The parent study consisted of a 6-week experience sampling with seven weekly laboratory sessions. After signing an informed consent form in the first lab session and after an introduction in the Android application movisensXS (movisens GmbH, Karlsruhe, Germany) that delivered the signals, participants received six randomly distributed signals each day for 40 subsequent days. These six signals were distributed between 10 am and 8 pm, with a mean interval of *M* = 103.4 min (*SD* = 34.3, range: 45–200 min). Participants returned to the laboratory each week, where they completed questionnaires and a computer-based guided-breathing meditation in the mindfulness training condition each week (see Rowland et al., 2016, 2019 for more information).

#### Measures

The descriptive statistics of the measures of Study 1 can be found in Table 2.

**Table 2:** Descriptive statistics (mean, standard deviation, within-/between-person reliability, intraclass correlation, and zero-order associations) of the variables in Study 1

Variable	*M*^a^	*SD*	*ω*_within_	*ω*_between_	ICC_person_	ICC_days|person_	1	2	3	4	5
1. Reappraisal	0.96	0.82	–	.99^b^	.36	.51	-	** − .56**	.09	**.34**	**.34**
2. Mindful attention (MAAS)	4.76	0.80	.80	.96	.44	.58	** − .14**	-	.16	** − .58**	** − .55**
3. PA	52.46	14.39	.81	.92	.43	.64	− .02	**.17**	-	** − .32**	** − .31**
4. NA	16.32	12.51	.71	.92	.47	.69	**.12**	** − .20**	** − .50**	-	**.67**
5. Depletion (SSCCS)	0.75	0.70	.79	.91	.40	.61	**.12**	** − .26**	** − .31**	**.35**	-

*ω*_*within*_ within-person reliability (McNeish, 2018), *ω*_*within*_ between-person reliability, *MAAS* Mindfulness Attention Awareness Scale, *PA* positive affect, *NA* negative affect, *SSCCS* State Self-Control Capacity Scale. Estimates in bold are significant at *p* < 0.05

Within-person correlations are shown in the lower left corner, and between-person correlations in the upper right corner

^a^The mean was calculated based on the person-level aggregated variables

^b^Spearman-Brown reliability coefficient between reappraisal scores aggregated on odd and even days (Eisinga et al., 2013)

##### Reappraisal

In response to each signal, participants indicated to what extent they have viewed the cause of their feelings from a different perspective since the last signal, using a scale from 1 (*not at all*) to 7 (*very much*). The item was adapted from Koval et al. (2015): “Since the last beep, I have viewed the cause of my feelings from a different perspective”. To compute the between-person reliability for single items, we split the data into odd and even days by coding the day variable (ranging from day 1 to day 40) with the sequence 0, 1, 0, 1, and so forth. We then aggregated the measures on the new variable for each individual and computed the Spearman-Brown reliability coefficient between the two test halves (Eisinga et al., 2013; see Table 2).

##### Mindful Attention

Mindful attention was assessed by selecting the three items from the state version of the Mindfulness Attention Awareness Scale (MAAS; Brown & Ryan, 2003) that yielded the highest factor loadings (Item 8: “I rush through activities without being really attentive to them.”; Item 10: “I do jobs or tasks automatically, without being aware of what I’m doing.”; Item 14: “I find myself doing things without paying attention.”). These items were rated on a scale ranging from 0 (*not at all*) to 6 (*very much*) and then inverted, so that higher mean scores indicate higher levels of mindful attention. Within-person reliability (Table 2) was estimated in accordance with Geldhof et al. (2014).

##### Affect

Current PA and NA were assessed at the moment of responding to the signal based on Kuppens et al. (2010), taking items from the circumplex model of affect (Russell, 1980). Each emotion item (Table 1) was rated on a scale ranging from 0 to 100, with higher values indicating stronger emotions.

##### Costs: Subjective Depletion

Subjective levels of depletion were assessed via three items from the State Self-Control Capacity Scale (SSCCS; Bertrams et al., 2011), which ranged from 1 (*not true*) to 7 (*very true*). The 3 items chosen were *I feel mentally exhausted*; *I want to give up*; and *I feel like my willpower is gone*.

#### Statistical Approach

For the within-person analyses, we computed multivariate 4-level models (Baldwin et al., 2014). The three outcome variables (PA, NA, and SSCCS) were simultaneously modeled at Level-1, which allows to test whether random slopes for different outcomes significantly correlate. These three outcome variables were nested within measurement occasions (observations at each signal, Level-2), nested within days (Level-3), and nested within participants (Level-4). For the between-person analyses, we aggregated each outcome on the person-level, resulting in a multivariate two-level model.

In the result section, the term *current* indicates the assessment of a variable at signal *t*, *prior* at signal *t*–1, and *recent* at the interval between the current assessment *t* and the prior assessment *t*–1.^2^ All variables termed current, prior, or recent were within-person standardized. We favored within-person standardization over within-person centering (Enders & Tofighi, 2007) since within-person standardization provides standard effect sizes, allowing easier interpretation and comparison of the effect sizes. Level-4 variables reflecting between-person differences were *z*-standardized. To test the difference between the associations of reappraisal and mindful attention when included in the same model, we computed the Wald test:

##### $z=\frac{{\beta }_{\mathrm{Reappraisal}}-{\beta }_{\mathrm{Mindful\ attention}}}{\sqrt{{{\mathrm{SE}}}_{\mathrm{Reappraisal}}^{2}+{{\mathrm{SE}}}_{\mathrm{Mindful\ attention}}^{2}+2*{\mathrm{cov}}_{\mathrm{Reappraisal},\mathrm{ Mindful\ attention}}}}$.

All analyses were performed using Stata 16 (Stata Corporation, College Station, TX).

###### Within-Person Analyses

No predictors at Level-3 (day level) were introduced to the four-level model as Level-3 was only introduced to improve model fit. As a preliminary analysis, we computed within-person associations reflecting zero-order associations. Following recent recommendations (Bakdash & Marusich, 2017), we computed the averaged within-person correlations by using 3-level models; in each of these, one within-person standardized variable (*y*) given an observation *i* nested in days *j* nested in participants *k* was regressed on the fixed (${\beta }_{11}$) and random, person-specific slope (${v}_{11k}^{x}$) of one Level-1 predictor (${x}_{ijk}$; see Eq. 1). Please note that we included the fixed intercept (${\beta }_{10}$), which was zero, and the random intercept (${\mu }_{1k}^{\mathrm{Days}|\mathrm{Participants}}$) of days but did not include a random intercept for participants (${\mu }_{2k}^{\mathrm{Participants}})$ given that we used within-person standardization, resulting in *M* = 0 for all variables and, thus, no differences in participants’ intercepts.

1 $${y}_{ijk}={\beta }_{10}+{\mu }_{1k}^{\mathrm{Days}|\mathrm{Participants}}+{(\beta }_{11}{+v}_{11k}){x}_{ijk}+{\varepsilon }_{ijk}$$

To test Hypotheses 1 to 3, we computed a single multivariate 4-level model (variables nested in observations nested in days nested in participants) where we regressed PA, NA, and depletion (SSCCS) on within-person standardized recent reappraisal (REA) and recent mindful attention (MIND). In addition, we included the two-way interactions between the *z*-standardized mindfulness training (MT) and reappraisal and mindful attention, respectively, to control for the possible influence of the mindfulness training (Eq. 2). Moreover, we controlled for prior PA (predicting only PA), prior NA (predicting only NA), and prior SSCCS (predicting only SSCCS) to examine the changes in the respective outcomes in the context of recent reappraisal and recent mindful attention.

2 $$\begin{array}{c}{y}_{hijk }={\beta }_{10}{\mathrm{PA}}_{k}+{\beta }_{20}{\mathrm{NA}}_{k}+{\beta }_{30}{\mathrm{SSCCS}}_{k}+{(\beta }_{11}+{v}_{11k}){\mathrm{REA}}_{ijk}{\mathrm{PA}}_{k}+{(\beta }_{21}+{v}_{21k}){\mathrm{REA}}_{ijk}{\mathrm{NA}}_{\mathrm{k}}+\\ {(\beta }_{31}+{v}_{31k}){\mathrm{REA}}_{ijk}{\mathrm{SSCCS}}_{k}+{(\beta }_{12}+{v}_{12k}){\mathrm{MIND}}_{ijk}{\mathrm{PA}}_{k}+{(\beta }_{22}+{v}_{22k}){\mathrm{MIND}}_{ijk}{\mathrm{NA}}_{k}+\\ \begin{array}{c}{(\beta }_{32}+{v}_{32k}){\mathrm{MIND}}_{ijk}{\mathrm{SSCCS}}_{k}+{\beta }_{13}{\mathrm{PriorPA}}_{ijk}{PA}_{k}+{\beta }_{23}{\mathrm{PriorNA}}_{ijk}{\mathrm{NA}}_{k}+\\ \begin{array}{c}{\beta }_{33}{\mathrm{PriorSSCCS}}_{ijk}{\mathrm{SSCCS}}_{k}+{\beta }_{14}{\mathrm{REA}\times \mathrm{MT}}_{ijk}{\mathrm{PA}}_{k}+{\beta }_{24}{\mathrm{REA}\times \mathrm{MT}}_{ijk}{\mathrm{NA}}_{k}+\\ \begin{array}{c}{\beta }_{34}{\begin{array}{c}REA\times \\ MT\end{array}}_{ijk}{\mathrm{SSCCS}}_{k}+{\beta }_{15}{\mathrm{MIND}\times \mathrm{MT}}_{ijk}{\mathrm{PA}}_{k}+{\beta }_{25}{\mathrm{MIND}\times \mathrm{MT}}_{ijk}{\mathrm{NA}}_{k}+\\ {\beta }_{35}{\mathrm{MIND}\times \mathrm{MT}}_{ijk}{\mathrm{SSCCS}}_{k}+{\mu }_{1jk}^{\mathrm{Days}|\mathrm{Participants}}+{\varepsilon }_{1ijk}{\mathrm{PA}}_{k}+{\varepsilon }_{2ijk}{\mathrm{NA}}_{k}+{\varepsilon }_{3ijk}{\mathrm{SSCCS}}_{k}\end{array}\end{array}\end{array}\end{array}$$

Unpacking Eq. 2, *y*_*hijk*_ is the respective outcome *h* (*y*_1*ijk*_ = PA, *y*_2*ijk*_ = NA, and *y*_3*ijk*_ = SSCCS) at time *i* of day *j* for person *k. β*_10_PA is the intercept of PA. PA_*k*_, NA_*k*_, and SSCCS_*k*_ are indicator variables: for example, PA_*k*_ is 1 for PA, 0 for NA, and 0 for SSCCS. Consequently, terms that include NA_*k*_ or SSCCS_*k*_ are not included in the prediction of PA (*y*_1*ijk*_). The term ${(\beta }_{11}+{v}_{11k}){\mathrm{REA}}_{ijk}{PA}_{k}$ is the fixed (${\beta }_{11}$) and random slope (${v}_{11k}$) of PA regressed on reappraisal. ${\beta }_{13}{\mathrm{PriorPA}}_{ijk}{\mathrm{PA}}_{k}$ controls for prior PA at time *t* − 1 and ${\beta }_{14}{\mathrm{REA}\times \mathrm{MT}}_{ijk}{\mathrm{PA}}_{k}$ is the two-way interaction between reappraisal and the mindful attention training on PA. Please note that we did not include the main fixed effect of the mindful attention training nor random person intercepts given that we within-person standardized the Level-2 variables including the outcome. Thus, each mean outcome was zero for each participant, leaving no random person intercept variance that could be explained by Level-4 predictors such as the mindfulness training. Moreover, we allowed for random covariances but only included random slopes for reappraisal and mindful attention to avoid convergence issues due to an increased complexity of an already complex multivariate multilevel model.

Following recent recommendations (de Haan-Rietdijk et al., 2016), we compared model fit using the Akaike Information Criterion (AIC) to test whether the inclusion of days as a level improved model fit. Given that this was the case in all models, we kept days as a level in all within-person analyses to avoid overestimating the size and significance of the within-person (Level-2) estimates and to consider variation between days.

###### Exploratory Between-Person Analyses

Our hypotheses were based on prior research on the within-person level (i.e., the level of variation from occasion to occasion). Accordingly, the analytical approach just described is of central relevance for this paper. Additionally, we provide results for the between-person level in an exploratory fashion (i.e., the level of aggregated states across study time, which reflect average tendencies in behavior). These might differ from the within-person level, as associations on one level of analysis do not necessarily translate to the other (Fisher et al., 2018). We could not examine between-person associations in the same model because we within-person standardized the within-person variables, which removes all between-person variance. Instead, we aggregated the measures on the person-level (Level-4) and regressed *z*-standardized PA, NA, and SSCCS on *z*-standardized aggregated reappraisal, mindful attention, and mindfulness training as well as on the two-way interactions between the mindfulness training and reappraisal and mindful attention in a multivariate two-level model where the outcomes were nested in participants (Eq. 3).

3 $$\begin{array}{c}{y}_{hk }={\beta }_{10}{\mathrm{PA}}_{k}+{\beta }_{20}{\mathrm{NA}}_{k}+{\beta }_{30}{\mathrm{SSCCS}}_{k}+{\beta }_{11}{\mathrm{REA}}_{k}{\mathrm{PA}}_{k}+{\beta }_{21}{\mathrm{REA}}_{k}{\mathrm{NA}}_{k}\\ +{\beta }_{31}{\mathrm{REA}}_{k}{\mathrm{SSCCS}}_{k}+{\beta }_{12}{\mathrm{MIND}}_{k}{\mathrm{PA}}_{k}+{\beta }_{22}{\mathrm{MIND}}_{k}{\mathrm{NA}}_{k}\\ \begin{array}{c}+{\beta }_{32}{\mathrm{MIND}}_{k}{\mathrm{SSCCS}}_{k}+{\beta }_{14}{\mathrm{REA}\times \mathrm{MT}}_{k}{\mathrm{PA}}_{k}+{\beta }_{24}{\mathrm{REA}\times \mathrm{MT}}_{k}{\mathrm{NA}}_{k}\\ \begin{array}{c}+{\beta }_{34}{\mathrm{REA}\times \mathrm{MT}}_{k}{\mathrm{SSCCS}}_{k}+{\beta }_{15}{\mathrm{MIND}\times \mathrm{MT}}_{k}{\mathrm{PA}}_{k}\\ \begin{array}{c}+{\beta }_{25}{\mathrm{MIND}\times \mathrm{MT}}_{k}{\mathrm{NA}}_{k}+{\beta }_{35}{\mathrm{MIND}\times \mathrm{MT}}_{k}{\mathrm{SSCCS}}_{k}+{\beta }_{16}{\mathrm{MT}}_{k}\\ +{\varepsilon }_{1k}{\mathrm{PA}}_{k}+{\varepsilon }_{2k}{\mathrm{NA}}_{k}+{\varepsilon }_{3k}{\mathrm{SSCCS}}_{k}\end{array}\end{array}\end{array}\end{array}$$

Although multivariate multilevel models have many benefits when comparing different outcomes (Baldwin et al., 2014), they were rarely computed in prior ER research. To facilitate comparison with evidence from prior research, we also performed univariate analyses, whose results can be found in Tables S7–S13 in the online supplementary material at OSF (https://osf.io/5kqnc). Please note that the fixed and random effects of the multivariate models were identical or very close to those of the univariate analyses with PA, NA, and SSCCS as the respective outcome.

### Results

#### Benefits of Reappraisal and Mindful Attention: Associations with PA and NA

As illustrated in Table 3 (the full results can be found at https://osf.io/dezrj/), the multivariate four-level model revealed that only recent mindful attention but not recent reappraisal was significantly associated with increases in current PA (Hypothesis 1): when individuals indicated being more mindful than usual, they also experienced more PA, which was not the case for reappraisal. Thus, against Hypothesis 1, the difference between reappraisal and mindful attention when testing the differences with a Wald test was significant but in the opposite direction to expectations, *b*_diff_ =  − 0.13, SE = 0.02, *p* < 0.001. That is, recent mindful attention was more strongly associated with increases in PA than reappraisal.

**Table 3:** Coefficients of reappraisal and mindful attention predicting positive affect, negative affect, and subjective depletion in Study 1

Outcome	Standardized regression coefficients		Wald test of the difference in regression coefficients
	Reappraisal	Mindful attention	Reappraisal–mindful attention
Benefits (ESM self-reports on the within-person level)			
PA	.02 [− 0.004, 0.04]	**.14** [0.12, 0.17]	− **0.13** [− 0.16, − 0.09]
NA	**.06** [0.04, 0.08]	− **.15** [− 0.18, − 0.12]	**0.21** [0.18, 0.24]
Depletion	**.05** [0.03, 0.08]	− **.22** [− 0.25, − 20]	**0.28** [0.24, 0.32]
Benefits (ESM self-reports on the between-person level)			
PA	**.30** [0.09, 0.51]	**.32** [0.12, 0.52]	− 0.02 [− 0.21, 0.18]
NA	.08 [− 0.09, 0.26]	− **.56** [− 0.73, − 0.38]	**0.64** [0.47, 0.80]
Depletion	.10 [− 0.07, 0.28]	− **.50** [− 0.68, − 0.33]	**0.61** [0.44, 0.77]

*ESM* experience sampling method, *PA* positive affect, *NA* negative affect. Estimates in bold are significant at *p* < 0.05

Regarding NA (Hypothesis 2), we found significant associations for both recent reappraisal and mindful attention on the within-person level, but in a different direction than expected (Table 3): Whereas being more mindful than usual was associated with *decreased* NA, endorsing more reappraisal than what an individual is typically endorsing on average was associated with *increased* NA. Given that the difference was significant and in the predicted direction, *b*_diff_ = 0.21, SE = 0.02, *p* < 0.001 (Table 3), we found support for Hypothesis 2.

For the between-person analyses, we aggregated the data on the person-level and then computed the multivariate two-level regression. In line with the within-person analyses, we found support for both Hypothesis 1 and 2: Both the aggregated means of reappraisal and of mindful attention were significantly associated with *higher* levels of PA, and the difference between the two predictors was not significant, indicating similar associations of reappraisal and mindful attention with PA (Hypothesis 1). For the aggregated means of NA, mindful attention but not reappraisal was significantly associated with *lower* levels of NA, with a significant difference in the predicted direction (Hypothesis 2).

Taken together, we found evidence for the predicted differential benefits of reappraisal and mindful attention but more clearly regarding NA and not PA: Compared to reappraisal, mindful attention was significantly more strongly associated with *less* NA, but, unexpectedly, also more strongly (at the within-person level) or as strongly (at the between-person level) with PA.

#### Costs of Reappraisal and Mindful Attention: Associations with Subjective Depletion

Regarding the costs of endorsing ER strategies, we hypothesized that reappraisal was associated with higher levels of subjective depletion than mindful attention (Hypothesis 3). The multivariate four-level model, indicated in Table 3, demonstrated that endorsing reappraisal more strongly than a participant was usually doing was significantly associated with an *increase* in subjective depletion. In contrast, recent mindful attention was associated with significantly *decreased* current subjective depletion. The difference between recent reappraisal and mindful attention was significant in the predicted direction (see Table 3).

The results on the between-person level were consistent with the within-person level: Whereas the aggregated means of reappraisal were *positively* and significantly associated with the aggregated means of subjective depletion, the aggregated means of mindful attention were *negatively* and significantly associated with the aggregated means of subjective depletion. Again, the difference was significant in the predicted direction.

#### Related Associations in Costs and Benefits

Finally, in addition to our hypotheses, we explored whether changes in costs due to the endorsement of reappraisal and mindful attention correlated with changes in benefits. This can be tested by examining the covariances between the random slopes of, for example, reappraisal in predicting the three outcomes. In the present research, we were interested in whether the associations of reappraisal and PA/NA were significantly related to the associations of reappraisal and subjective depletion. Thus, do individuals with a larger increase in NA after endorsing reappraisal also experience larger increases in subjective depletion after endorsing reappraisal? And indeed, the multivariate four-level model for Hypotheses 1 to 3 revealed that *increases *in NA when endorsing reappraisal were positively coupled with *increases* in subjective depletion when endorsing reappraisal, *b*_cov_ = 0.005, SE = 0.002, *p* = 0.021, 95% CI [0.001, 0.009], and *r* = 0.35, revealing a medium effect size. No significant association was found for PA, *b*_cov_ =  − 0.002, SE = 0.002, *p* = 0.231, 95% CI [− 0.005, 0.001], and *r* =  − 0.21. These couplings were larger for mindful attention: *Decreases* in NA when endorsing mindful attention were positively coupled with *decreases* in subjective depletion when in a mindful state, *b*_cov_ = 0.009, SE = 0.002, *p* < 0.001, 95% CI [0.005, 0.014], and *r* = 0.64. Moreover, *increases in PA* when endorsing mindful attention were coupled with *decreases* in subjective depletion, *b*_cov_ =  − 0.006, SE = 0.002, *p* = 0.011, 95% CI [− 0.011, − 0.001], and *r* =  − 0.36. However, the covariances of the absolute value of the random slopes did not differ significantly between reappraisal and mindful attention, neither for the PA-SSCCS association, *b*_diff_ =  − 0.004, SE = 0.003, and *p* = 0.143, nor for the NA-SSCCS association, *b*_diff_ = 0.005, SE = 0.003, and *p* = 0.132. Thus, costs and benefits were similarly associated when participants endorsed reappraisal or were being mindful, which did not provide evidence for the notion that the lower costs of endorsing mindful attention were significantly more strongly associated with greater benefits.

#### Strength of Endorsing Reappraisal and Mindful Attention

Finally, we tested Hypothesis 4 that mindful attention is favored over reappraisal. To that end, we aggregated the reported endorsement of reappraisal and mindful attention on the person-level and computed a two-level model in which the aggregated values of reappraisal and mindful attention were nested within individuals. We controlled for the mindfulness training by including the main effect of the mindfulness training and its two-way interaction with the binary variable (0 = reappraisal, 1 = mindfulness). This model revealed that reappraisal was endorsed less strongly in daily life (*M* = 0.96, SE = 0.07) than mindful attention (*M* = 4.76, SE = 0.07), *t*(123) =  − 35.94, *p* < 0.001, and *d*_*z*_ =  − 3.24, which provides support for Hypothesis 4.

#### Alternative Explanations

Moreover, we tested the alternative explanation that reappraisal is associated with greater costs in terms of subjective depletion than mindful attention because it is more strongly endorsed when participants experience strong NA and feelings of depletion. To that end, we computed a mixed model, in which we predicted either reappraisal or mindful attention by prior NA and prior subjective depletion, controlling for prior endorsement of reappraisal or mindful attention. This model revealed that prior NA was associated with an *increased* endorsement of reappraisal, *β* = 0.05, SE = 0.01, *p* < 0.001, 95% [0.03, 0.07], but with a *decreased* endorsement of mindful attention, *β* =  − 0.03, SE = 0.01, *p* < 0.001, 95% [− 0.05, − 0.01]. Moreover, prior subjective depletion was also significantly associated with a *decreased* endorsement of mindful attention, *β* =  − 0.06, SE = 0.01, *p* < 0.001, 95% [− 0.08, − 0.04], although it was not significantly associated with increased reappraisal, *β* = 0.02, SE = 0.01, *p* = 0.136, 95% [− 0.01, 0.04]. Thus, reappraisal might be associated with more subjective depletion because it is endorsed in response to more severe NA than mindful attention. To test this alternative explanation, we included prior NA in the analyses for testing the costs of reappraisal and mindful attention in terms of subjective depletion (Hypothesis 3) and found that the results did not change meaningfully: The coefficient of reappraisal changed from *β* = 0.06 to *β* = 0.05 and was still significant, *p* < 0.001. The coefficient of mindful attention did not change and was still medium in size, *β* =  − 0.22, *p* < 0.001. Thus, we could not find evidence for this alternative explanation: Although reappraisal was endorsed significantly more strongly for higher levels of NA, the increased costs of reappraisal in terms of subjective depletion compared to mindful attention remained the same when controlling for prior NA. However, future research could systematically explore the interplay between beneficial associations in ER and the insights participants have into them to unlock the full potential of ER.^3^

Another alternative explanation for the finding that mindful attention compared to reappraisal was less costly could be that by practicing it in the mindfulness condition, participants in the mindfulness training condition became more effective in endorsing a mindful state. To test this alternative explanation, we computed a mixed model in which within-person standardized subjective depletion was predicted by within-person standardized mindful attention, mindfulness training, and block (0 = first 2 weeks of the ESM; 1 = last 2 weeks of the ESM) along with all two-way and three-way interactions between either reappraisal or mindful attention with the mindfulness training and block, controlling for prior subjective depletion at *t* − 1. This model did not yield a significant three-way interaction between mindful attention, mindfulness training, and block, *z* = 0.26, *p* = 0.795. Looking at the simple slopes revealed that participants in the mindfulness training condition did not show a larger negative association between mindful attention and subjective depletion in the last (*β* =  − 0.21, SE = 0.02, *p* < 0.001, 95% [− 0.26, − 0.16]) compared to the first 2 weeks of the ESM (*β* =  − 0.21, SE = 0.03, *p* < 0.001, 95% [− 0.26, − 0.15]), *b* = 0.004, SE = 0.03, *p* = 0.880, 95% [− 0.05, 0.06]. The three-way interaction between reappraisal, mindfulness training, and block was also not significant, *z* = 0.59, *p* = 0.555, and the two-interactions between mindfulness training and either mindful attention or reappraisal were not significant as well. Thus, we can rule out the alternative explanation that the negative association between mindful attention and costs was driven by the mindfulness training.

### Discussion

Study 1 looked at the benefits and costs of reappraisal and mindful attention. Our hypotheses were largely supported: Compared to mindful attention, reappraisal was less strongly related to decreases in NA (Hypothesis 2) and was significantly associated with increases in subjective depletion (Hypothesis 3) and lower endorsement (Hypothesis 4). Of note, regarding Hypothesis 2, reappraisal was significantly and positively related to increases in NA. This may suggest that efforts to use reappraisal to combat increases in NA (e.g., following stressors) were ineffective. Regarding Hypothesis 1, where we only could test one part of the hypothesis in Study 1 that reappraisal and mindful attention were similarly associated with PA, we only found support on the between-person level but not on the within-person level. Here, mindful attention was significantly more strongly associated with increased PA than reappraisal.

Finally, the results from the multivariate multilevel models show that changes in NA and depletion in the context of either mindfulness or reappraisal were positively associated. Individuals who reported less subjective depletion in the context of mindful attention also reported lower levels of NA (*r* = .64). It might thus be that benefits in terms of decreasing NA when endorsing mindful attention can be achieved because mindful attention has relatively low costs when being endorsed. Changes in NA and depletion were also positively associated for reappraisal (*r* = .35): Individuals who reported more subjective depletion also reported higher levels of NA. Thus, it might be that potential costs of reappraisal in terms of depletion contribute to the finding that NA is enhanced in the context of reappraisal. Finally, the correlations just reported did not differ significantly from each other, although the NA-depletion coupling was twice as large when participants were in a more mindful state (*r* = .64, Fisher’s z = 0.74) than when they endorsed reappraisal (*r* = .35, Fisher’s z = 0.37). The fact that this relatively large difference did not reach significance despite Study 1’s sample with a relatively large number of observations might be due to the low power of these tests (Hertzog et al., 2006). Thus, given the correlational nature of our approach, additional research based on studies with more power is needed to further determine the causal ordering of any of these effects.

Taken together, our results demonstrate possible differential costs and benefits of reappraisal and mindful attention in daily life. However, given that in Study 1 only mindful attention was measured with the unidimensional MAAS, we were not able to differentiate between the mindfulness components, which might explain the diverging results regarding Hypothesis 1. While the MAAS nominally measures mindfulness attention and awareness, mindful acceptance as an additional defining characteristic of mindfulness was found to be relevant in research on within-person variation in mindfulness in daily life (Bergomi et al., 2013; Blanke et al., 2018). Accordingly, the aims of Study 2 were to (a) replicate the evidence regarding benefits and (b) differentiate between different the two mindfulness components mindful attention and mindful acceptance.

## Study 2

### Method

Table 1 provides an overview of the dataset in Study 2. In contrast to Study 1, participants in Study 2 were not subjected to a mindfulness training.

#### Participants

The target sample size of the parent study of 180 participants was almost achieved with *N* = 179 participants (52.5% female, *M*_age_ 50.9 years, *SD*_age_ = 5.8). Participants could receive up to 90 €, with 10 € contingent on completing at least 60 observations in the experience sampling phase of the study.

#### Procedure

Participants were visited in their private households to sign an informed consent form, complete questionnaires, and receive the study smartphones (Huawei Ascend G33) including an introduction on how to use them. The experience sampling started the following day and was broken up into three assessment phases: each phase consisting of four sampling days with a pause of up to 4 days between the phases. Two of the four pausing days were optionally used to prolong the assessment phase if participants missed more than one assessment a day. Adherence was very good, with 98.7% completed signals consistent with prior publications. All data were analyzed, including signals that were only partially completed.

#### Measures

Descriptive information and reliabilities are reported in Table 4.

**Table 4:** Descriptive statistics (mean, standard deviation, within-/between-person reliability, intraclass correlation, and zero-order associations) of the state measures in Study 2

State measure	*M*^a^	*SD*	*ω*_within_	*ω*_between_	ICC_person_	ICC_days|person_	1	2	3	4	5
1. Reappraisal	2.45	1.41	–	.87^b^	.49	.59	-	− .13	**.15**	**.37**	.04
2. Mindful acceptance	4.52	1.13	–	.79^b^	.42	.57	− .03	-	.12	**.18**	** − .70**
3. Mindful attention	3.79	1.21	–	.84^b^	.45	.56	**.07**	**.05**	-	**.42**	** − .25**
4. PA	3.19	0.76	.81	.91	.39	.59	**.16**	**.14**	**.21**	-	** − .29**
5. NA	1.05	0.81	.81	.96	.47	.63	** − **.02	** − .33**	** − .16**	** − .37**	-

*ω*_within _within-person reliability and *ω*_between_ between-person reliability (Geldhof et al., 2014; McNeish, 2018). *PA *positive affect, *NA *negative affect

^a^The mean was calculated based on the person-level aggregated variables. Estimates in bold are significant at *p* < 0.05

^b^To compute the between-person reliability for single items, we computed the mean for each of the three measurement bursts and then used the three indicators in a structural equation model to compute *ω*_between_

Averaged within-person correlations are shown in the lower left corner, and between-person correlations in the upper right corner

##### Reappraisal

Reappraisal was assessed as positive reappraisal using the item “Since the last signal/Since waking up: I’ve been looking for the positive side of the matter” (original wording: *Seit der letzten Befragung/ Seit dem Aufwachen: Ich habe nach positiven Seiten der Angelegenheit gesucht*), using a response scale ranging from 0 (*not at all*) to 6 (*very much*).

##### Mindful Attention and Acceptance

Mindful acceptance and attention were assessed by one item each that were based on the Multidimensional State Mindfulness Questionnaire (MSMQ; Blanke & Brose, 2017). The item for mindful acceptance was “Things went through my mind that I should not really be engaging myself with” (reverse coded; original wording: *Mir sind Dinge durch den Kopf gegangen, die mich eigentlich nicht beschäftigen sollten*) and for mindful attention “I opened myself up to what was happening (e.g., a meal/conversation/music)” (original wording: *Ich habe mich auf das eingelassen, was gerade geschah [z.B. ein Essen/ein Gespräch/Musik]*). Both items were rated on a scale ranging from 0 (*not at all*) to 6 (*very much*) and, like reappraisal, referred to the time between beeps/since waking up.

##### Affect

Current PA and NA were measured using six items each selected to represent higher and lower arousal in accordance with the quadrants of the affect circumplex (Russell, 2003), including PANAS items (Watson et al., 1988). All affect items (Table 1) were rated on a scale ranging from 0 (*not at all*) to 6 (*very much*).

#### Analytic Approach

The analytic approach in Study 2 was the same as in Study 1.

### Results

#### Benefits of Reappraisal and Mindfulness: Associations with Positive and Negative Affect

We first were interested in the differential relation between change in affect from one measurement occasion to the next and recent reappraisal, mindful acceptance, and mindful attention to examine benefits of these strategies. To that end, we computed multivariate four-level models (measures nested within observations nested within days nested within individuals) predicting current PA and NA by recent reappraisal, recent mindful attention, and recent mindful acceptance while controlling for prior NA and PA and allowing for random slopes and covariances (as in Study 1). Table 5 shows that recent reappraisal, mindful acceptance, and mindful attention were all significantly associated with increases in PA. Importantly, the Wald test showed that the difference between the coefficient of recent reappraisal and of mindful acceptance testing Hypothesis 1 was significant and in the predicted direction, but that the difference between the coefficient of recent reappraisal and of mindful attention was not significant. Thus, reappraisal and mindful attention were both more strongly associated with increases in PA than mindful acceptance, which demonstrates the adaptiveness of reappraisal over mindful acceptance, but not mindful attention, in terms of upregulating PA.

**Table 5:** Coefficients of person-level aggregated reappraisal, mindful attention, and mindful acceptance predicting well-being on the within- and between-person level in Study 2

Outcome	Standardized regression coefficients			Wald test of the difference in regression coefficients		
	Reappraisal	Mindful acceptance	Mindful attention	Reappraisal–mindful acceptance	Reappraisal–mindful attention	Acceptance–mindful attention
Within-person level benefits (ESM self-reports on the within-person level)						
PA	**.13** [0.11, 0.15]	**.08** [0.05, 0.11]	**.14** [0.12, 0.17]	**0.05** [0.01, 0.09]	− 0.01 [− 0.05, 0.03]	** − 0.06** [− 0.10, − 0.02]
NA	− .02 [− 0.05, 0.01]	** − .26** [− 0.30, − 0.23]	** − .12** [− 0.15, − 0.09]	**0.25** [0.20, 0.29]	**0.10** [0.06, 0.14]	** − 0.14** [− 0.19, − 0.10]
Between-person level benefits (ESM self-reports on the between-person level)						
PA	**.35** [0.23, 0.48]	.12 [− 0.004, 0.25]	**.33** [0.21, 0.46]	**0.23** [0.06, 0.39]	0.02 [− 0.18, 0.21]	** − 0.21** [− 0.40, − 0.02]
NA	− .02 [− 0.13, 0.08]	** − .68** [− 0.79, − 0.58]	** − .16** [-0.27, − 0.06]	**0.66** [0.52, 0.80]	0.14, [− 0.02, 0.30]	** − 0.52** [− 0.68, − 0.37]

*ESM* experience sampling method, *PA* positive affect, *NA* negative affect

In turn, recent mindful acceptance and mindful attention were both significantly related to decreases in NA, whereas recent reappraisal was not. To test Hypothesis 2, that mindful acceptance was more effective in decreasing NA than reappraisal and mindful attention, we computed Wald tests again, which yielded support for Hypothesis 2: Endorsing mindful acceptance in daily life was significantly associated with a stronger decrease in NA compared to endorsing reappraisal and mindful attention (Table 5).

To examine between-person differences in the relation between affect, reappraisal, mindful acceptance, and mindful attention, we computed a multivariate two-level model predicting person-aggregated PA and NA by person-aggregated reappraisal, mindful attention, and mindful acceptance. As illustrated in Table 5, the results were consistent with the within-person analyses, demonstrating that individuals who endorsed reappraisal more strongly than others reported higher levels of PA. Testing Hypothesis 1 by computing Wald tests revealed that the difference between the coefficient of aggregated reappraisal and aggregated mindful acceptance was significant and in the predicted direction, *b* = 0.23, SE = 0.08, and *p* = 0.006. The difference between the coefficient of aggregated reappraisal and aggregated mindful attention, however, was not significant. In turn, individuals who accepted their own thoughts and feelings more strongly than others, but not those who endorsed reappraisal more strongly than others, reported lower levels of NA. Regarding Hypothesis 2, we found that mindful acceptance was significantly more strongly associated with decreases in NA than reappraisal and mindful attention, *b* = 0.66, SE = 0.07, and *p* < 0.001, with no significant difference between the latter (Table 5).

Thus, our results suggest a differential relation between changes in affect and the regulatory efforts of reappraisal, mindful attention, and mindful acceptance.

#### Strength of Endorsing Reappraisal, Mindful Acceptance, and Mindful Attention

Based on prior research, we hypothesized that reappraisal was endorsed less strongly than mindful acceptance or mindful attention (Hypothesis 4). As in Study 1, reappraisal was endorsed less strongly (*M* = 2.45, *SD* = 1.41) compared to mindful acceptance (*M* = 4.52, *SD* = 1.13), *t*(178) =  − 14.48, *p* < 0.001, and *d*_*z*_ =  − 1.08, or mindful attention (*M* = 3.79, *SD* = 1.21), *t*(178) =  − 10.47, *p* < 0.001, and *d*_*z*_ =  − 0.78. Finally, mindful acceptance was endorsed more strongly than mindful attention, *t*(178) =  − 6.32, *p* < 0.001, and *d*_*z*_ =  − 0.47.

### Discussion

In Study 2, we replicated our results in relation to Hypotheses 1, 2, and 4. Additionally, we differentiated between the mindfulness facets mindful attention and mindful acceptance to compare their associations with affective well-being in relation to reappraisal to fully test Hypotheses 1 and 2. In line with Hypothesis 1, we found that reappraisal and mindful attention were both similarly associated with PA, suggesting that reappraising a situation and being aware of momentary sensations can be equally more beneficial for one’s present PA than accepting things as they are. Moreover, both reappraisal and mindful attention were significantly more strongly associated with increased PA than mindful acceptance, providing support for Hypothesis 1 on the within- and between-person level.

In line with Hypothesis 2, we found that mindful acceptance was significantly more strongly associated with decreased NA than reappraisal and mindful attention. This was the case not only on the within- but also on the between-person level. In line with the possible benefits of mindful acceptance, individuals also preferred endorsing mindful acceptance most intensely in daily life, while reappraisal was endorsed less intensely than mindful attention and acceptance, which confirmed Hypothesis 4.

In sum, the findings in Study 2 suggest (i) that it is important to examine the unique effects of mindfulness facets on present affective experiences, (ii) that associations between reappraisal/mindfulness and affective well-being were consistent on the within- and between-person level, and (iii) that there was not a single strategy that was generally (most) adaptive for both PA and NA. Instead, mindful acceptance was most adaptive for decreasing NA, whereas reappraisal and mindful attention were most effective in increasing PA.

## General Discussion

Regarding the benefits, we found that positive reappraisal and mindful attention were equally associated with increases in PA, while mindful acceptance was more strongly associated with decreases in NA. Concerning the costs of their spontaneous use, our findings show that when individuals endorsed cognitive reappraisal more strongly, they also felt more depleted. Given that mindful attention was associated with less subjective depletion, we suppose that the endorsement of mindfulness was mentally less taxing, replicating past laboratory studies’ findings that mindful acceptance is less effortful to endorse than reappraisal (Troy et al., 2018) and may help to restore cognitive resources (Friese et al., 2012). This also connects to research showing that individuals are often unsuccessful in reappraising a negative situation in daily life (Ford et al., 2017), which can be detrimental for one’s mental health (Ford et al., 2017; Ford & Troy, 2019) and which may explain the positive association between reappraisal and NA in Study 1. In line with this finding, we found that individuals endorsed mindfulness more intensely than reappraisal, which might suggest that they prefer beneficial, but less mentally taxing approaches to ER.

PA and particularly NA were strongly correlated with subjective depletion when endorsing either reappraisal or mindfulness: Participants who decreased their NA more strongly by ER also reported stronger decreases in subjective depletion. Thus, individuals who were less compared to more depleted also reported to experience increased PA or decreased NA, hinting at the importance of the costs of ER for individuals’ affective well-being. Thus, our findings demonstrate that ER research should not only focus on the benefits associated with ER strategies but also acknowledge their costs to better understand when ER efforts fail.

In the present study, we investigated the predictive value of each approach (mindful attention and acceptance, as well as reappraisal) for affective well-being above and beyond the other approaches. However, previous research also suggests that these approaches may be used in combination and potentially sequentially. For example, the Monitoring and Acceptance Theory (e.g., Lindsay & Creswell, 2017) assumes that mindful attention and acceptance need to interact to improve well-being. Furthermore, although mindfulness and reappraisal may seem like opposite processes, this may not the case: The mindfulness-to-meaning theory (e.g., Garland et al., 2015) proposes that mindfulness leads to more positive reappraisal, with research showing that mindfulness-based interventions can improve not only mindfulness but also positive emotion regulation skills such as reappraisal (Guendelman et al., 2017; Jennings et al., 2017). Moreover, other mindfulness facets than mindful attention and acceptance (that other conceptualizations of mindfulness entail), such as decentering, may also be relevant here. Others have also argued that mindful awareness to internal processes, such as thoughts and feelings, without avoidance may foster flexibility in choosing appropriate strategies, which may very well entail changing emotions in turn (Alkoby et al., 2019). We therefore think that future studies should not only consider the cost and benefits of different approaches to emotion regulation in isolation, but should also include how different strategies are effectively combined to achieve well-being.

There are also other limitations to our study: First, we found in Study 1 that when individuals endorsed reappraisal more intensely than usual, they also reported more NA. This finding may be explained by the different wordings of the reappraisal items: In Study 1, reappraisal was measured by asking individuals to what degree they have tried to view the cause of their feelings from a different perspective, which is best characterized as *cognitive reappraisal*. In Study 2, individuals were asked to what degree they have tried to view the positive side of a matter, which is an operationalization of *positive reappraisal* (Garland et al., 2009). Thus, unlike the item in Study 2, the item in Study 1 does not necessarily aim at improving present feelings, which is in line with a past study’s finding: Bringing to mind the possible causes of an event was found to maintain anger, whereas positive reappraisal and distraction reduced it (Denson et al., 2012).

Second, our data was not specifically collected to answer our present research questions. Therefore, mindfulness in Study 1 was assessed using three items from the MAAS (Brown & Ryan, 2003), which is a unidimensional measure of attention/awareness. However, it has been argued that the MAAS, in its full 15-item version, also captures nonjudgmental mindful acceptance by using solely negatively formulated items that implicitly reflect a judging stance (Bergomi et al., 2013). And indeed, at the between-person level, the MAAS is more strongly associated with the acceptance subscale of other mindfulness questionnaires than with an observing stance (e.g., Höfling et al., 2011). Thus, we cannot clearly state whether mindful attention or acceptance or both restore one’s cognitive capacities and are mentally less taxing than reappraisal. Relatedly, the assessment of mindfulness in Study 2 was also not optimal because the mindfulness facets were measured with single items. This impedes estimating reliability and aggregation and thus likely leads to an underestimation of effects. Furthermore, we were not able to investigate Hypothesis 3 in Study 2, as it did not include a measure of depletion.

Fourth, we cannot make any causal conclusions based on our results. Thus, there may be other factors possibly contributing to the association between reappraisal and subjective depletion. For example, it may be the case that individuals already tried other strategies to regulate their feelings before endorsing reappraisal (Guiller et al., 2019), which may be mentally taxing by itself. Moreover, individuals may not freely adopt strategies but instead may react to situational changes that trigger endorsing the respective strategy.

Fifth, the participants in both datasets were not asked whether they needed to regulate their emotions. Thus, we could not disentangle whether the lowest value in reappraisal endorsement (i.e., not at all) was selected due to a lack of attempting to engage in ER or due not wanting to endorse reappraisal specifically.

Finally, one important difference in the spontaneous use of emotion regulation strategies in daily life compared to the instructed use in laboratory settings is that the latter often involves a controlled stimulus which participants are instructed to regulate. Change in affect before and after the stimulus captures the effectiveness of a particular emotion regulation strategy. However, in daily life, much may happen between two measurement occasions and, thus, the target of ER attempts is unclear. This might explain, for example, the positive association of reappraisal and increases in NA in Study 1—negative events since the last measurement occasion might still have been lingering at the current measurement occasion. Future research may address this issue by using experimental manipulation of emotion regulation strategies or by examining the context in which emotion regulation takes place in greater detail than what has been commonly done in experience sampling research (but without overburdening the participants given the complexity of the context and environment in daily life).

## Conclusion

We provide evidence for the costs and benefits associated with endorsing reappraisal and mindfulness. While reappraisal and mindful attention were equally associated with effective upregulation of PA, mindful acceptance was more strongly associated with effective downregulation of NA. Given that individuals endorsed mindfulness more strongly than reappraisal and that the latter was more mentally taxing, they may prefer beneficial, but less mentally taxing strategies in daily life. Consequently, accepting emotions as they are instead of trying to change them may be a healthy and comparatively less cognitively effortful way to handle difficult situations in everyday life. Future studies may further explore how both approaches may potentially be combined to achieve well-being.

## Supplementary Information

Below is the link to the electronic supplementary material.

Supplementary file1 (DOCX 99 KB)

## Footnotes

1 In the following, we use the term mindfulness when a measure contains multiple components or when it refers to the concept as a whole and not to individual components.

2 Given that the items used to assess reappraisal and mindfulness asked individuals how much they have engaged in mindful attention or reappraisal since the last signal, these items capture what happened between the successive time points *t* and *t* − 1. In this case, we call these variables “recent,” even though they were assessed at the current timepoint *t*.

3 We also tested whether, in addition to the main associations between reappraisal or mindfulness and PA and NA, their interaction was associated with changes in PA and NA, which would speak for the idea that associations of emotion regulation may vary as a function of affect intensity. However, this was not the case, all *p*s > .079.

## Additional Information

We affirm that we reported all manipulations and exclusions in the present study but not all measures of the parent studies that recruited the samples.

### Data and Code Availability

Dataset 1 as well as all statistical analyses and the full results can be found at OSF (https://osf.io/dezrj/). This dataset is from the SMASH study, which examined the effectiveness of a mindfulness intervention (Rowland et al., 2016, 2019). Dataset 2 is a study that was implemented within the Innovation Sample of the German Socio-Economic Panel (SOEP-IS, Richter & Schupp, 2015). The study is Wave 1 of the “Everyday Experiences” in the SOEP-IS study (EE-SOEP-IS; Blanke et al., 2020; Siebert et al., 2017). The data can be obtained via the German Institute for Economic Research (DIW, 2020), whereas all statistical analyses and the full results can be found at OSF (https://osf.io/dezrj/).
