# Supplementary material for: The Costs and Benefits of Mindfulness and Reappraisal in Daily Life
Source: Affect Sci. 2023 Feb 20;4(2):260–74. doi: 10.1007/s42761-022-00178-7 (PMC10247578; doi:10.1007/s42761-022-00178-7)
Supplement: Supplementary file 1 — Supplementary file1 (DOCX 99 KB) [file 42761_2022_178_MOESM1_ESM.docx]

**Tables**

Table S1

*Fixed-effects parameters of the multivariate four-level model in which reappraisal and mindfulness predicted current positive affect, negative affect, and subjective depletion in Study 1, testing Hypothesis 1 to 3 on the within-person level*

| **Fixed-effects parameters** | **β** | ***SE*** | ***z*** | ***p*** | **ll** | **ul** |
| --- | --- | --- | --- | --- | --- | --- |
| Reappraisal (PA) | .015 | .010 | 1.50 | .134 | -.005 | .035 |
| Reappraisal (NA) | .060 | .013 | 4.75 | <.001 | .035 | .085 |
| Reappraisal (SSCCS) | .054 | .014 | 3.94 | <.001 | .027 | .080 |
| Mindfulness (PA) | .143 | .014 | 10.09 | <.001 | .115 | .171 |
| Mindfulness (NA) | -.150 | .013 | -11.64 | <.001 | -.175 | -.125 |
| Mindfulness (SSCCS) | -.223 | .014 | -15.91 | <.001 | -.250 | -.195 |
| MT | .001 | .003 | 0.23 | .817 | -.006 | .007 |
| Reappraisal (PA) x MT | -.002 | .010 | -0.18 | .860 | -.022 | .018 |
| Reappraisal (NA) x MT | -.001 | .013 | -0.08 | .933 | -.026 | .024 |
| Reappraisal (SSCCS) x MT | -.009 | .014 | -0.66 | .510 | -.036 | .018 |
| Mindfulness (PA) x MT | .004 | .014 | 0.28 | .782 | -.024 | .032 |
| Mindfulness (NA) x MT | .011 | .013 | 0.84 | .402 | -.014 | .036 |
| Mindfulness (SSCCS) x MT | .005 | .014 | 0.33 | .745 | -.023 | .032 |
| Prior PA (PA) | .396 | .006 | 61.09 | <.001 | .383 | .409 |
| Prior NA (NA) | .393 | .006 | 61.69 | <.001 | .381 | .406 |
| Prior SSCCS (SSCCS) | .325 | .007 | 46.23 | <.001 | .311 | .339 |
| Intercept PA | .016 | .007 | 2.24 | .025 | .002 | .029 |
| Intercept NA | .005 | .007 | 0.78 | .436 | -.008 | .019 |
| Intercept SSCCS | .031 | .007 | 4.33 | <.001 | .017 | .045 |

*Note.* PA = positive affect; NA = negative affect; SSCCS = depletion; MT = Mindfulness training, ll = lower bound of the 95% confidence interval; ul = upper bound of the 95% confidence interval. The variable in the brackets refer to the respective outcome.

Table S2

*Random-effects parameters of the multivariate four-level model in which reappraisal and mindfulness predicted current positive affect, negative affect, and subjective depletion in Study 1, testing Hypothesis 1 to 3 on the within-person level*

| **Random-effects parameters** | **Var** | ***SE*** | ***z*** | ***p*** | **ll** | **ul** |
| --- | --- | --- | --- | --- | --- | --- |
| Reappraisal (PA) | .006 | .002 | 3.41 | .001 | .003 | .010 |
| Reappraisal (NA) | .012 | .003 | 4.74 | <.001 | .008 | .018 |
| Reappraisal (SSCCS) | .015 | .003 | 5.09 | <.001 | .010 | .021 |
| Mindfulness (PA) | .017 | .003 | 5.44 | <.001 | .012 | .025 |
| Mindfulness (NA) | .013 | .003 | 4.94 | <.001 | .009 | .020 |
| Mindfulness (SSCCS) | .016 | .003 | 5.14 | <.001 | .011 | .024 |
| Reappraisal (PA) x Reappraisal (NA) | -.003 | .002 | -1.95 | .052 | -.006 | >-.001 |
| Reappraisal (PA) x Reappraisal (SSCCS) | -.002 | .002 | -1.20 | .231 | -.005 | .001 |
| Reappraisal (PA) x Mindfulness (PA) | >-.001 | .002 | -0.14 | .886 | -.003 | .003 |
| Reappraisal (PA) x Mindfulness (NA) | -.002 | .001 | -1.09 | .277 | -.004 | .001 |
| Reappraisal (PA) x Mindfulness (SSCCS) | -.003 | .002 | -1.98 | .048 | -.006 | >-.001 |
| Reappraisal (NA) x Reappraisal (SSCCS) | .005 | .002 | 2.31 | .021 | .001 | .009 |
| Reappraisal (NA) x Mindfulness (PA) | -.007 | .002 | -3.44 | .001 | -.012 | -.003 |
| Reappraisal (NA) x Mindfulness (NA) | .003 | .002 | 1.74 | .082 | >-.001 | .007 |
| Reappraisal (NA) x Mindfulness (SSCCS) | -.001 | .002 | -0.40 | .687 | -.005 | .003 |
| Reappraisal (SSCCS) x Mindfulness (PA) | -.003 | .002 | -1.46 | .143 | -.007 | .001 |
| Reappraisal (SSCCS) x Mindfulness (NA) | .001 | .002 | 0.37 | .712 | -.003 | .005 |
| Reappraisal (SSCCS) x Mindfulness (SSCCS) | -.002 | .002 | -0.85 | .395 | -.006 | .002 |
| Mindfulness (PA) x Mindfulness (NA) | -.011 | .002 | -4.42 | <.001 | -.016 | -.006 |
| Mindfulness (PA) x Mindfulness (SSCCS) | -.006 | .002 | -2.54 | .011 | -.011 | -.001 |
| Mindfulness (NA) x Mindfulness (SSCCS) | .009 | .002 | 4.03 | <.001 | .005 | .014 |
| Intercept Days | .001 | .001 | 1.02 | .307 | ->.001 | .008 |
| Residual PA | .752 | .009 | 86.68 | <.001 | .736 | .770 |
| Residual NA | .739 | .009 | 86.47 | <.001 | .723 | .756 |
| Residual SSCCS | .785 | .009 | 86.43 | <.001 | .767 | .803 |
| Residual PA x Residual NA | -.337 | .007 | -49.64 | <.001 | -.350 | -.324 |
| Residual PA x Residual SSCCS | -.204 | .007 | -30.86 | <.001 | -.217 | -.191 |
| Residual NA x Residual SSCCS | .221 | .007 | 33.83 | <.001 | .208 | .234 |

*Note.* PA = positive affect; NA = negative affect; SSCCS = depletion; MT = Mindfulness training, ll = lower bound of the 95% confidence interval; ul = upper bound of the 95% confidence interval; Var = Variance. The variable in the brackets refer to the respective outcome.

Table S3

*Fixed- and random-effects parameters of the multivariate two-level model in which reappraisal and mindfulness predicted current positive affect, negative affect, and subjective depletion in Study 1, testing Hypothesis 1 to 3 on the between-person level*

| **Random-effects parameters** | **β** | ***SE*** | ***z*** | ***p*** | **ll** | **ul** |
| --- | --- | --- | --- | --- | --- | --- |
| Reappraisal (PA) | .303 | .108 | 2.81 | .005 | .092 | .514 |
| Reappraisal (NA) | .083 | .090 | 0.91 | .360 | -.094 | .259 |
| Reappraisal (SSCCS) | .104 | .091 | 1.14 | .252 | -.074 | .283 |
| Mindfulness (PA) | .320 | .104 | 3.08 | .002 | .117 | .524 |
| Mindfulness (NA) | -.555 | .087 | -6.40 | <.001 | -.725 | -.385 |
| Mindfulness (SSCCS) | -.504 | .088 | -5.75 | <.001 | -.676 | -.332 |
| MT | -.072 | .042 | -1.73 | .084 | -.154 | .010 |
| Reappraisal (PA) x MT | -.056 | .109 | -0.51 | .609 | -.269 | .158 |
| Reappraisal (NA) x MT | -.165 | .091 | -1.81 | .070 | -.344 | .014 |
| Reappraisal (SSCCS) x MT | -.113 | .092 | -1.23 | .219 | -.293 | .067 |
| Mindfulness (PA) x MT | -.020 | .104 | -0.19 | .847 | -.224 | .184 |
| Mindfulness (NA) x MT | .001 | .087 | 0.01 | .990 | -.170 | .172 |
| Mindfulness (SSCCS) x MT | .116 | .088 | 1.32 | .187 | -.056 | .288 |
| Intercept PA | .010 | .088 | 0.12 | .905 | -.162 | .183 |
| Intercept NA | .040 | .074 | 0.55 | .584 | -.104 | .185 |
| Intercept SSCCS | .046 | .074 | 0.62 | .538 | -.100 | .191 |
| **Random-effects parameters** | **Var** | ***SE*** | ***z*** | ***p*** | **ll** | **ul** |
| Residual PA | .908 | .115 | 7.91 | <.001 | .708 | 1.163 |
| Residual NA | .635 | .080 | 7.89 | <.001 | .495 | .814 |
| Residual SSCCS | .647 | .082 | 7.90 | <.001 | .505 | .829 |
| Residual PA x Residual NA | -.231 | .071 | -3.26 | .001 | -.370 | -.092 |
| Residual PA x Residual SSCCS | -.244 | .072 | -3.39 | .001 | -.385 | -.103 |
| Residual NA x Residual SSCCS | .320 | .064 | 4.99 | <.001 | .194 | .446 |

*Note.* PA = positive affect; NA = negative affect; SSCCS = depletion; MT = Mindfulness training, ll = lower bound of the 95% confidence interval; ul = upper bound of the 95% confidence interval; Var = Variance. The variable in the brackets refer to the respective outcome.

Table S4

*Fixed-effects parameters of the multivariate two-level model in which reappraisal, acceptance, and present-moment attention predicted current positive affect and negative affect in Study 2, testing Hypothesis 1b and 2b on the within-person level*

| **Fixed-effects parameters** | **β** | ***SE*** | ***z*** | ***p*** | **ll** | **ul** |
| --- | --- | --- | --- | --- | --- | --- |
| Reappraisal (PA) | .131 | .012 | 10.76 | <.001 | .107 | .155 |
| Reappraisal (NA) | -.018 | .015 | -1.19 | .235 | -.048 | .012 |
| Acceptance (PA) | .083 | .014 | 5.79 | <.001 | .055 | .110 |
| Acceptance (NA) | -.264 | .017 | -15.79 | <.001 | -.297 | -.231 |
| Attention (PA) | .144 | .014 | 10.60 | <.001 | .117 | .171 |
| Attention (NA) | -.120 | .014 | -8.85 | <.001 | -.146 | -.093 |
| Prior PA (PA) | .245 | .009 | 27.58 | <.001 | .228 | .263 |
| Prior NA (NA) | .222 | .009 | 24.64 | <.001 | .205 | .240 |
| Intercept PA | .022 | .009 | 2.51 | .012 | .005 | .040 |
| Intercept NA | -.018 | .009 | -2.04 | .042 | -.036 | -.001 |

*Note.* PA = positive affect; NA = negative affect; SSCCS = depletion; MT = Mindfulness training, ll = lower bound of the 95% confidence interval; ul = upper bound of the 95% confidence interval; Var = variance. The variable in the brackets refer to the respective outcome.

Table S5

*Random-effects parameters of the multivariate two-level model in which reappraisal, acceptance, and present-moment attention predicted current positive affect and negative affect in Study 2, testing Hypothesis 1b and 2b on the within-person level*

| **Random-effects parameters** | **Var** | ***SE*** | ***z*** | ***p*** | **ll** | **ul** |
| --- | --- | --- | --- | --- | --- | --- |
| Reappraisal (PA) | .011 | .003 | 4.08 | <.001 | .007 | .018 |
| Reappraisal (NA) | .025 | .004 | 5.72 | <.001 | .018 | .036 |
| Acceptance (PA) | .020 | .004 | 5.31 | <.001 | .014 | .029 |
| Acceptance (NA) | .032 | .005 | 6.21 | <.001 | .023 | .044 |
| Attention (PA) | .017 | .003 | 4.92 | <.001 | .011 | .025 |
| Attention (NA) | .016 | .003 | 4.70 | <.001 | .011 | .025 |
| Reappraisal (PA) x Reappraisal (NA) | -.004 | .003 | -1.40 | .163 | -.008 | .001 |
| Reappraisal (PA) x Acceptance (PA) | -.003 | .002 | -1.17 | .243 | -.007 | .002 |
| Reappraisal (PA) x Acceptance (NA) | -.001 | .003 | -0.27 | .789 | -.006 | .005 |
| Reappraisal (PA) x Attention (PA) | -.005 | .002 | -2.50 | .013 | -.010 | -.001 |
| Reappraisal (PA) x Attention (NA) | .004 | .002 | 1.99 | .047 | <.001 | .009 |
| Reappraisal (NA) x Acceptance (PA) | -.008 | .003 | -2.70 | .007 | -.014 | -.002 |
| Reappraisal (NA) x Acceptance (NA) | -.003 | .003 | -0.84 | .400 | -.009 | .004 |
| Reappraisal (NA) x Attention (PA) | .005 | .003 | 1.89 | .058 | >-.001 | .011 |
| Reappraisal (NA) x Attention (NA) | -.001 | .003 | -0.40 | .686 | -.006 | .004 |
| Acceptance (PA) x Acceptance (NA) | >-.001 | .003 | -0.14 | .886 | -.007 | .006 |
| Acceptance (PA) x Attention (PA) | -.002 | .002 | -1.01 | .311 | -.007 | .002 |
| Acceptance (PA) x Attention (NA) | -.009 | .003 | -3.37 | .001 | -.014 | -.004 |
| Acceptance (NA) x Attention (PA) | -.003 | .003 | -0.97 | .333 | -.009 | .003 |
| Acceptance (NA) x Attention (NA) | -.001 | .003 | -0.45 | .654 | -.007 | .004 |
| Attention (PA) x Attention (NA) | -.006 | .003 | -2.32 | .021 | -.011 | -.001 |
| Intercept Days | .013 | .003 | 4.45 | <.001 | .008 | .020 |
| Residual PA | .677 | .011 | 64.24 | <.001 | .657 | .698 |
| Residual NA | .684 | .011 | 64.70 | <.001 | .664 | .705 |
| Residual PA x Residual NA | -.158 | .008 | -20.34 | <.001 | -.173 | -.143 |

*Note.* PA = positive affect; NA = negative affect; SSCCS = depletion; MT = Mindfulness training, ll = lower bound of the 95% confidence interval; ul = upper bound of the 95% confidence interval; Var = Variance. The variable in the brackets refer to the respective outcome.

Table S6

*Fixed- and random-effects parameters of the multivariate two-level model in which reappraisal, acceptance, and present-moment attention predicted current positive affect and negative affect in Study 2, testing Hypothesis 1b and 2b on the between-person level*

| **Fixed-effects parameters** | **β** | ***SE*** | ***z*** | ***p*** | **ll** | **ul** |
| --- | --- | --- | --- | --- | --- | --- |
| Reappraisal (PA) | .351 | .065 | 5.43 | <.001 | .224 | .478 |
| Reappraisal (NA) | -.024 | .053 | -0.46 | .649 | -.128 | .080 |
| Acceptance (PA) | .122 | .064 | 1.90 | .058 | -.004 | .248 |
| Acceptance (NA) | -.684 | .053 | -12.95 | <.001 | -.788 | -.581 |
| Attention (PA) | .334 | .065 | 5.16 | <.001 | .207 | .461 |
| Attention (NA) | -.161 | .053 | -3.04 | .002 | -.265 | -.057 |
| Intercept PA | .000 | .063 | 0.00 | 1.000 | -.124 | .124 |
| Intercept NA | .000 | .052 | 0.00 | 1.000 | -.101 | .101 |
| **Random-effects parameters** | **Var** | ***SE*** | ***z*** | ***p*** | **ll** | **ul** |
| Residual PA | .711 | .075 | 9.46 | <.001 | .578 | .875 |
| Residual NA | .478 | .051 | 9.46 | <.001 | .389 | .589 |
| Residual PA x Residual NA | -.044 | .044 | -1.02 | .309 | -.130 | .041 |

*Note.* PA = positive affect; NA = negative affect; SSCCS = depletion; MT = Mindfulness training, ll = lower bound of the 95% confidence interval; ul = upper bound of the 95% confidence interval; Var = Variance. The variable in the brackets refer to the respective outcome.

Table S7

*Fixed- and random-effects parameters of the univariate three-level model in which reappraisal and mindfulness predicted current positive affect in Study 1, testing Hypothesis 1 on the within-person level*

| **Fixed-effects parameters** | **β** | ***SE*** | ***z*** | ***p*** | **ll** | **ul** |
| --- | --- | --- | --- | --- | --- | --- |
| Reappraisal | .017 | .009 | 1.80 | .071 | -.001 | .035 |
| Mindfulness | .139 | .014 | 9.72 | <.001 | .111 | .167 |
| Reappraisal x MT | -.004 | .009 | -0.41 | .684 | -.022 | .014 |
| Mindfulness x MT | <.001 | .014 | 0.02 | .982 | -.027 | .028 |
| Prior PA | .394 | .016 | 24.03 | <.001 | .362 | .426 |
| Intercept | .015 | .007 | 2.01 | .044 | <.001 | .029 |
| **Random-effects parameters** | **Var** | ***SE*** | ***z*** | ***p*** | **ll** | **ul** |
| Reappraisal | .004 | .001 | 2.66 | .008 | .002 | .008 |
| Mindfulness | .018 | .003 | 5.40 | <.001 | .012 | .026 |
| Prior PA | .026 | .004 | 6.30 | <.001 | .019 | .036 |
| Reappraisal ~ mindfulness | -.001 | .001 | -0.60 | .549 | -.004 | .002 |
| Reappraisal ~ prior PA | -.002 | .002 | -1.06 | .291 | -.005 | .002 |
| Mindfulness ~ prior PA | -.005 | .003 | -1.74 | .082 | -.010 | .001 |
| Day intercept | .036 | .008 | 4.29 | <.001 | .023 | .057 |
| Residual | .693 | .011 | 64.31 | <.001 | .672 | .714 |

*Note.* PA = positive affect; MT = Mindfulness training, ll = lower bound of the 95% confidence interval; ul = upper bound of the 95% confidence interval; Var = Variance.

Table S8

*Fixed- and random-effects parameters of the univariate three-level model in which reappraisal and mindfulness predicted current negative affect in Study 1, testing Hypothesis 2 on the within-person level*

| **Fixed-effects parameters** | **β** | ***SE*** | ***z*** | ***p*** | **ll** | **ul** |
| --- | --- | --- | --- | --- | --- | --- |
| Reappraisal | .057 | .012 | 4.61 | <.001 | .033 | .081 |
| Mindfulness | -.148 | .013 | -11.70 | <.001 | -.173 | -.124 |
| Reappraisal x MT | .001 | .012 | 0.12 | .906 | -.023 | .025 |
| Mindfulness x MT | .011 | .012 | 0.88 | .379 | -.013 | .035 |
| Prior NA | .403 | .018 | 22.57 | <.001 | .368 | .438 |
| Intercept | .006 | .007 | 0.86 | .389 | -.008 | .020 |
| **Random-effects parameters** | **Var** | ***SE*** | ***z*** | ***p*** | **ll** | **ul** |
| Reappraisal | .011 | .002 | 4.59 | <.001 | .007 | .017 |
| Mindfulness | .013 | .003 | 4.84 | <.001 | .008 | .019 |
| Prior NA | .032 | .005 | 6.61 | <.001 | .024 | .044 |
| Reappraisal ~ mindfulness | .002 | .002 | 0.87 | .385 | -.002 | .005 |
| Reappraisal ~ prior NA | -.002 | .002 | -1.01 | .312 | -.007 | .002 |
| Mindfulness ~ prior NA | .005 | .003 | 2.05 | .040 | <.001 | .010 |
| Day intercept | .018 | .008 | 2.35 | .019 | .008 | .041 |
| Residual | .692 | .011 | 65.19 | <.001 | .671 | .713 |

*Note.* NA = negative affect; MT = Mindfulness training, ll = lower bound of the 95% confidence interval; ul = upper bound of the 95% confidence interval; Var = Variance.

Table S9

*Fixed- and random-effects parameters of the univariate three-level model in which reappraisal and mindfulness predicted current subjective depletion in Study 1, testing Hypothesis 3 on the within-person level*

| **Fixed-effects parameters** | **β** | ***SE*** | ***z*** | ***p*** | **ll** | **ul** |
| --- | --- | --- | --- | --- | --- | --- |
| Reappraisal | .053 | .014 | 3.90 | <.001 | .026 | .079 |
| Mindfulness | -.222 | .014 | -15.72 | <.001 | -.250 | -.195 |
| Reappraisal x MT | -.008 | .013 | -0.58 | .565 | -.034 | .019 |
| Mindfulness x MT | .006 | .014 | 0.45 | .649 | -.021 | .033 |
| Prior SSCCS | .312 | .017 | 18.23 | <.001 | .278 | .345 |
| Intercept | .031 | .007 | 4.16 | <.001 | .016 | .046 |
| **Random-effects parameters** | **Var** | ***SE*** | ***z*** | ***p*** | **ll** | **ul** |
| Reappraisal | .014 | .003 | 5.03 | <.001 | .010 | .021 |
| Mindfulness | .017 | .003 | 5.16 | <.001 | .011 | .024 |
| Prior SSCCS | .028 | .005 | 6.17 | <.001 | .021 | .039 |
| Reappraisal ~ mindfulness | -.002 | .002 | -0.78 | .436 | -.006 | .002 |
| Reappraisal ~ prior SSCCS | <.001 | .003 | 0.04 | .966 | -.005 | .005 |
| Mindfulness ~ prior SSCCS | .006 | .003 | 2.11 | .035 | <.001 | .011 |
| Day intercept | .031 | .008 | 3.83 | <.001 | .018 | .051 |
| Residual | .731 | .011 | 66.66 | <.001 | .709 | .752 |

*Note.* SSCCS = depletion; MT = Mindfulness training, ll = lower bound of the 95% confidence interval; ul = upper bound of the 95% confidence interval; Var = Variance.

Table S10

*Parameters of the univariate regression models in which reappraisal and mindfulness predicted either person-aggregated positive affect, negative affect, or subjective depletion in Study 1, testing Hypothesis 1-3 on the between-person level*

| **Parameters** | **β** | ***SE*** | ***z*** | ***p*** | **ll** | **ul** |
| --- | --- | --- | --- | --- | --- | --- |
| Positive affect |  |  |  |  |  |  |
| Reappraisal | .304 | .112 | 2.71 | .008 | .082 | .526 |
| Mindfulness | .320 | .106 | 3.01 | .003 | .109 | .531 |
| MT | -.076 | .091 | -0.84 | .405 | -.255 | .104 |
| Reappraisal x MT | -.056 | .112 | -0.50 | .617 | -.278 | .165 |
| Mindfulness x MT | -.020 | .107 | -0.19 | .851 | -.231 | .191 |
| Intercept | .011 | .090 | 0.12 | .908 | -.168 | .189 |
| Negative affect |  |  |  |  |  |  |
| Reappraisal | .069 | .094 | 0.74 | .462 | -.116 | .254 |
| Mindfulness | -.554 | .089 | -6.24 | <.001 | -.730 | -.378 |
| MT | -.018 | .076 | -0.24 | .813 | -.168 | .132 |
| Reappraisal x MT | -.161 | .093 | -1.73 | .086 | -.346 | .023 |
| Mindfulness x MT | .003 | .089 | 0.03 | .975 | -.174 | .179 |
| Intercept | .040 | .075 | 0.53 | .598 | -.109 | .189 |
| Subjective depletion |  |  |  |  |  |  |
| Reappraisal | .116 | .094 | 1.23 | .220 | -.071 | .304 |
| Mindfulness | -.505 | .090 | -5.64 | <.001 | -.683 | -.328 |
| MT | -.122 | .076 | -1.60 | .113 | -.273 | .029 |
| Reappraisal x MT | -.116 | .094 | -1.24 | .219 | -.303 | .070 |
| Mindfulness x MT | .114 | .090 | 1.27 | .206 | -.064 | .292 |
| Intercept | .046 | .076 | 0.61 | .544 | -.104 | .197 |

*Note.* MT = Mindfulness training, ll = lower bound of the 95% confidence interval; ul = upper bound of the 95% confidence interval.

Table S11

*Fixed- and random-effects parameters of the univariate three-level model in which reappraisal, nonjudgmental acceptance, and present-moment attention predicted current positive affect in Study 2, testing Hypothesis 1b on the within-person level*

| **Fixed-effects parameters** | **β** | ***SE*** | ***z*** | ***p*** | **ll** | **ul** |
| --- | --- | --- | --- | --- | --- | --- |
| Reappraisal | .131 | .012 | 10.73 | <.001 | .107 | .155 |
| Acceptance | .082 | .014 | 5.89 | <.001 | .055 | .110 |
| Attention | .144 | .013 | 10.82 | <.001 | .118 | .170 |
| Prior PA | .235 | .013 | 17.65 | <.001 | .209 | .261 |
| Intercept | .022 | .009 | 2.35 | .019 | .004 | .040 |
| **Random-effects parameters** | **Var** | ***SE*** | ***z*** | ***p*** | **ll** | **ul** |
| Reappraisal | .011 | .003 | 4.04 | <.001 | .007 | .018 |
| Acceptance | .018 | .004 | 5.05 | <.001 | .012 | .027 |
| Attention | .015 | .003 | 4.61 | <.001 | .010 | .023 |
| Prior PA | .016 | .003 | 4.82 | <.001 | .011 | .024 |
| Reappraisal ~ acceptance | -.003 | .002 | -1.46 | .146 | -.007 | .001 |
| Reappraisal ~ attention | -.005 | .002 | -2.35 | .019 | -.009 | -.001 |
| Reappraisal ~ prior PA | <.001 | .002 | 0.23 | .814 | -.004 | .005 |
| Acceptance ~ attention | -.002 | .002 | -1.04 | .298 | -.007 | .002 |
| Acceptance ~ prior PA | -.001 | .002 | -0.40 | .692 | -.006 | .004 |
| Attention ~ prior PA | -.003 | .002 | -1.31 | .191 | -.007 | .001 |
| Day intercept | .032 | .009 | 3.73 | <.001 | .019 | .054 |
| Residual | .647 | .012 | 53.98 | <.001 | .624 | .671 |

*Note.* PA = positive affect, ll = lower bound of the 95% confidence interval; ul = upper bound of the 95% confidence interval; Var = Variance.

Table S12

*Fixed- and random-effects parameters of the univariate three-level model in which reappraisal, nonjudgmental acceptance, and present-moment attention predicted current negative affect in Study 2, testing Hypothesis 2b on the within-person level*

| **Fixed-effects parameters** | **β** | ***SE*** | ***z*** | ***p*** | **ll** | **ul** |
| --- | --- | --- | --- | --- | --- | --- |
| Reappraisal | -.016 | .015 | -1.04 | .298 | -.045 | .014 |
| Acceptance | -.261 | .017 | -15.67 | <.001 | -.294 | -.229 |
| Attention | -.114 | .014 | -8.42 | <.001 | -.141 | -.088 |
| Prior NA | .237 | .014 | 16.34 | <.001 | .208 | .265 |
| Intercept | -.019 | .009 | -2.19 | .029 | -.036 | -.002 |
| **Random-effects parameters** | **Var** | ***SE*** | ***z*** | ***p*** | **ll** | **ul** |
| Reappraisal | .024 | .004 | 5.64 | <.001 | .017 | .034 |
| Acceptance | .032 | .005 | 6.13 | <.001 | .023 | .044 |
| Attention | .016 | .003 | 4.71 | <.001 | .011 | .025 |
| Prior NA | .021 | .004 | 5.48 | <.001 | .015 | .031 |
| Reappraisal ~ acceptance | -.004 | .003 | -1.10 | .272 | -.010 | .003 |
| Reappraisal ~ attention | -.001 | .003 | -0.57 | .569 | -.007 | .004 |
| Reappraisal ~ prior NA | -.005 | .003 | -1.68 | .094 | -.010 | .001 |
| Acceptance ~ attention | -.001 | .003 | -0.18 | .857 | -.006 | .005 |
| Acceptance ~ prior NA | .005 | .003 | 1.41 | .158 | -.002 | .011 |
| Attention ~ prior NA | .003 | .003 | 0.99 | .324 | -.003 | .008 |
| Day intercept | .002 | .008 | 0.23 | .815 | <.001 | 7.816 |
| Residual | .675 | .013 | 53.64 | <.001 | .650 | .700 |

*Note.* NA = negative affect, ll = lower bound of the 95% confidence interval; ul = upper bound of the 95% confidence interval; Var = Variance.

Table S13

*Parameters of the univariate regression models in which reappraisal, nonjudgmental acceptance, and present-moment attention predicted either person-aggregated positive affect or negative affect in Study 2, testing Hypothesis 1b and 2b on the between-person level*

| **Parameters** | **β** | ***SE*** | ***z*** | ***p*** | **ll** | **ul** |
| --- | --- | --- | --- | --- | --- | --- |
| Positive affect |  |  |  |  |  |  |
| Reappraisal | .351 | .065 | 5.37 | <.001 | .222 | .480 |
| Acceptance | .122 | .065 | 1.87 | .063 | -.006 | .251 |
| Attention | .334 | .065 | 5.11 | <.001 | .205 | .463 |
| Intercept | 0 | .064 | 0 | 1 | -.126 | .126 |
| Negative affect |  |  |  |  |  |  |
| Reappraisal | -.024 | .054 | -0.45 | .653 | -.130 | .082 |
| Acceptance | -.684 | .053 | -12.80 | <.001 | -.790 | -.579 |
| Attention | -.161 | .054 | -3.00 | .003 | -.267 | -.055 |
| Intercept | 0 | .052 | 0 | 1 | -.103 | .103 |

*Note.* ll = lower bound of the 95% confidence interval; ul = upper bound of the 95% confidence interval.
